# Clinical use of artificial intelligence products for radiology in the Netherlands between 2020 and 2022

**DOI:** 10.1007/s00330-023-09991-5

**Published:** 2023-07-29

**Authors:** Kicky G. van Leeuwen, Maarten de Rooij, Steven Schalekamp, Bram van Ginneken, Matthieu J. C. M. Rutten

**Affiliations:** 1 Department of Medical Imaging, Radboud University Medical Center, Nijmegen, The Netherlands; 2 Department of Radiology, Jeroen Bosch Hospital, ’s-Hertogenbosch, The Netherlands

**Keywords:** Artificial intelligence, Radiology, Software, Medical informatics, Delivery of health care

## Abstract

**Objectives:**

To map the clinical use of CE-marked artificial intelligence (AI)–based software in radiology departments in the Netherlands (*n* = 69) between 2020 and 2022.

**Materials and methods:**

Our AI network (one radiologist or AI representative per Dutch hospital organization) received a questionnaire each spring from 2020 to 2022 about AI product usage, financing, and obstacles to adoption. Products that were not listed on www.AIforRadiology.com by July 2022 were excluded from the analysis.

**Results:**

The number of respondents was 43 in 2020, 36 in 2021, and 33 in 2022. The number of departments using AI has been growing steadily (2020: 14, 2021: 19, 2022: 23). The diversity (2020: 7, 2021: 18, 2022: 34) and the number of total implementations (2020: 19, 2021: 38, 2022: 68) has rapidly increased. Seven implementations were discontinued in 2022. Four hospital organizations said to use an AI platform or marketplace for the deployment of AI solutions. AI is mostly used to support chest CT (17), neuro CT (17), and musculoskeletal radiograph (12) analysis. The budget for AI was reserved in 13 of the responding centers in both 2021 and 2022. The most important obstacles to the adoption of AI remained costs and IT integration. Of the respondents, 28% stated that the implemented AI products realized health improvement and 32% assumed both health improvement and cost savings.

**Conclusion:**

The adoption of AI products in radiology departments in the Netherlands is showing common signs of a developing market. The major obstacles to reaching widespread adoption are a lack of financial resources and IT integration difficulties.

**Clinical relevance statement:**

The clinical impact of AI starts with its adoption in daily clinical practice. Increased transparency around AI products being adopted, implementation obstacles, and impact may inspire increased collaboration and improved decision-making around the implementation and financing of AI products.

**Key Points:**

*• The adoption of artificial intelligence products for radiology has steadily increased since 2020 to at least a third of the centers using AI in clinical practice in the Netherlands in 2022.*

*• The main areas in which artificial intelligence products are used are lung nodule detection on CT, aided stroke diagnosis, and bone age prediction.*

*• The majority of respondents experienced added value (decreased costs and/or improved outcomes) from using artificial intelligence–based software; however, major obstacles to adoption remain the costs and IT-related difficulties.*

**Supplementary Information:**

The online version contains supplementary material available at 10.1007/s00330-023-09991-5.

## Introduction

The field of radiology is a frontrunner in healthcare considering the commercially available artificial intelligence (AI)–based products [1]. There are now more than two hundred CE-marked AI products for radiology on the market for clinical use [2]. Vendors providing these products make claims on improving efficiency and quality of care [3], but to date, it remains unclear if these claims are being fulfilled in clinical practice. However, the first step towards creating an impact with AI in radiology is the adoption of these products.

Some research on individual radiologist’s experience and attitudes towards AI have been conducted both in the United States and in Europe [4–7]. However, there is no research on the adoption of AI on a per-practice base nor on the specific products that were adopted.

In this study, we monitored the clinical use of commercially available AI software in radiology departments in the Netherlands over a 3-year period (2020–2022). Also addressed are the experienced added value of AI software, discontinuance of AI products, platform adoption, the methods of financing AI, and the major obstacles to AI implementation.

## Materials and methods

### Data collection

No institutional review board approval was needed for this study. At the beginning of 2020, a communication network, the so-called AI network was established together with the Dutch Society of Radiology. This AI network has one radiologist per hospital organization that serves as a contact person regarding AI. In some hospital organizations, we were referred to healthcare professionals other than radiologists (e.g. clinical physicist, technical physician, application specialist, team manager), which were our additional contacts. The Netherlands has 69 hospital organizations of which 7 academic centers, 25 teaching hospitals, and 37 general hospitals [8]. We were able to collect contact details for 54 of the organizations. For three consecutive years (2020–2022), a questionnaire using Google Forms (Google LLC) was sent out to this group in February–March.

### Questionnaire

The main goal of the survey was to inquire about the clinical use of AI products for radiology. Apart from requesting the commercial AI products used in clinical practice (open), we asked about their intention to implement AI software (categorical), whether the budget was reserved for it in the upcoming year (categorical), and the obstacles for procurement, validation, and implementation of AI software (open).

In the last survey (2022), we added a question on the use of AI platforms or so-called marketplaces (open) and whether product implementations were terminated (open). Also, we asked whether the respondents considered the clinical use of AI to increase health and/or reduce costs (categorical).

### Analysis

As the definition of an AI product is debatable, only products that were listed on www.AIforRadiology.com [2], a listing of CE-marked AI software for radiology, by July 2022 were included. This comprised a total of 202 products provided by 97 vendors.

Open answers to the obstacles experienced were standardized to a set of topics that appeared more than once. The topics were defined based on the answers to the 2020 survey and were kept constant for the other years. One answer could address multiple topics. Topics were ‘Costs, lack of budget’, ‘Unclear business case’, ‘Diffuse supply’, ‘IT and integration’, ‘Lack of validation’, ‘Legal issues’, ‘Lack of vision, policy, ownership’. All other comments were captured as ‘Other’.

## Results

### Respondents

The number of respondents was 43 in 2020, 36 in 2021, and 33 in 2022 with a representative mix of academic, teaching, and general hospitals as demonstrated in Table 1. The majority of respondents were radiologists as presented in Table 2.

**Table 1:** Use of AI per hospital type as reported by the respondents

Respondents per hospital type	Total number of respondents			Respondents using AI			% of respondents using AI			% of total hospitals using AI		
	2020	2021	2022	2020	2021	2022	2020	2021	2022	2020	2021	2022
Academic hospital (*n* = 7)	5	5	6	3	4	4	60%	80%	67%	43%	57%	57%
Teaching hospital (*n* = 25)	15	15	16	7	8	14	47%	53%	88%	28%	32%	56%
General hospital (*n* = 37)	23	16	11	4	7	5	17%	44%	45%	11%	19%	14%
Total (*n* = 69)	43	36	33	14	19	23	33%	53%	70%	20%	28%	33%

**Table 2:** Respondents’ roles

Respondents’ role	2020	2021	2022
Radiologist	30	30	24
Management	3	2	0
Application specialist	5	1	2
Clinical physicist/clinical informatics/technical physician	3	2	5
Other	2	1	2

### Clinical use of AI

The desire to adopt AI in the radiology department of the respective center as reported by the respondents increased from 63% in 2020 to 86% and 88% in 2021 and 2022, respectively.

**Fig. 1:**
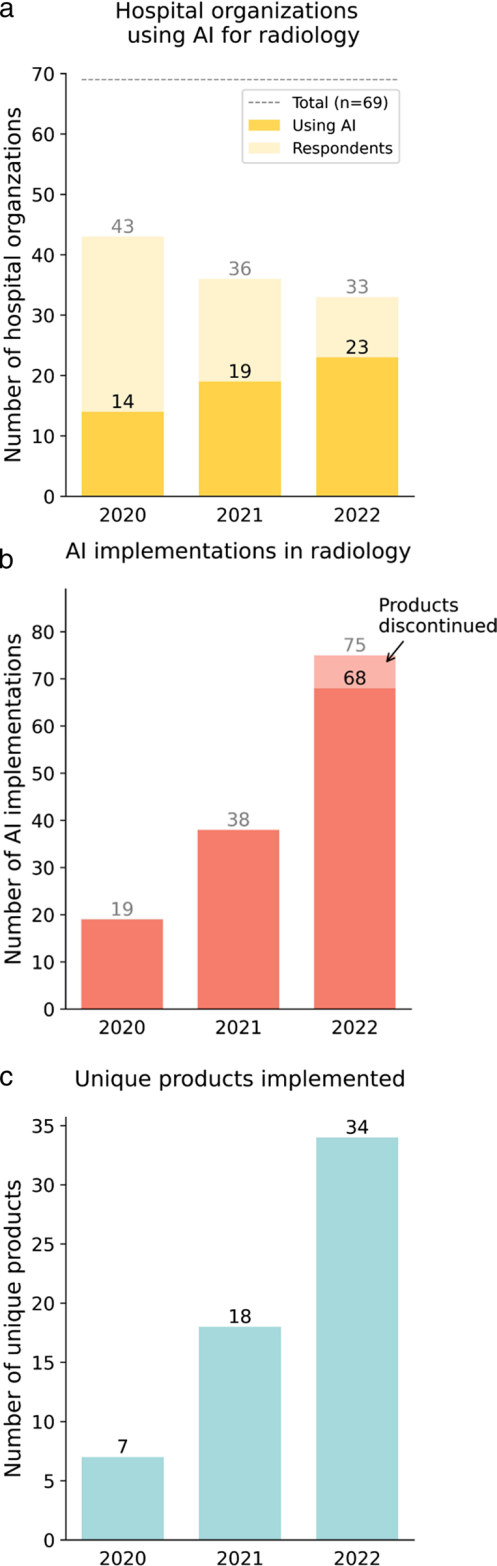
Clinical use of commercial AI in radiology departments in the Netherlands. **a** Hospital organizations using AI in their radiology department. **b** The total number of AI implementations. **c** The number of unique AI products being deployed in the Netherlands

Figure 1 and Table 1 show the use of AI products in clinical practice. The number of departments that were using one of the AI products as listed on www.AIforRadiology.com in clinical practice steadily grew from 14 (20%, 2020) to 19 (28%, 2021) to 23 (33%, 2022). The diversity of products implemented in the Netherlands increased by fivefold from seven unique products in 2020 to 34 in 2022. The cumulative number of AI implementations was 19 in 2020, 38 in 2021, and 68 in 2022. In other words, departments using AI had on average one AI product running in 2020, two in 2021, and three in 2022. In 2022, six departments were reported to have discontinued the use of in total seven products. Four hospital organizations said to use an AI platform or marketplace for the deployment of AI solutions of which three use it currently for the deployment of multiple AI solutions.

### Applications and reported value of AI

**Fig. 2:**
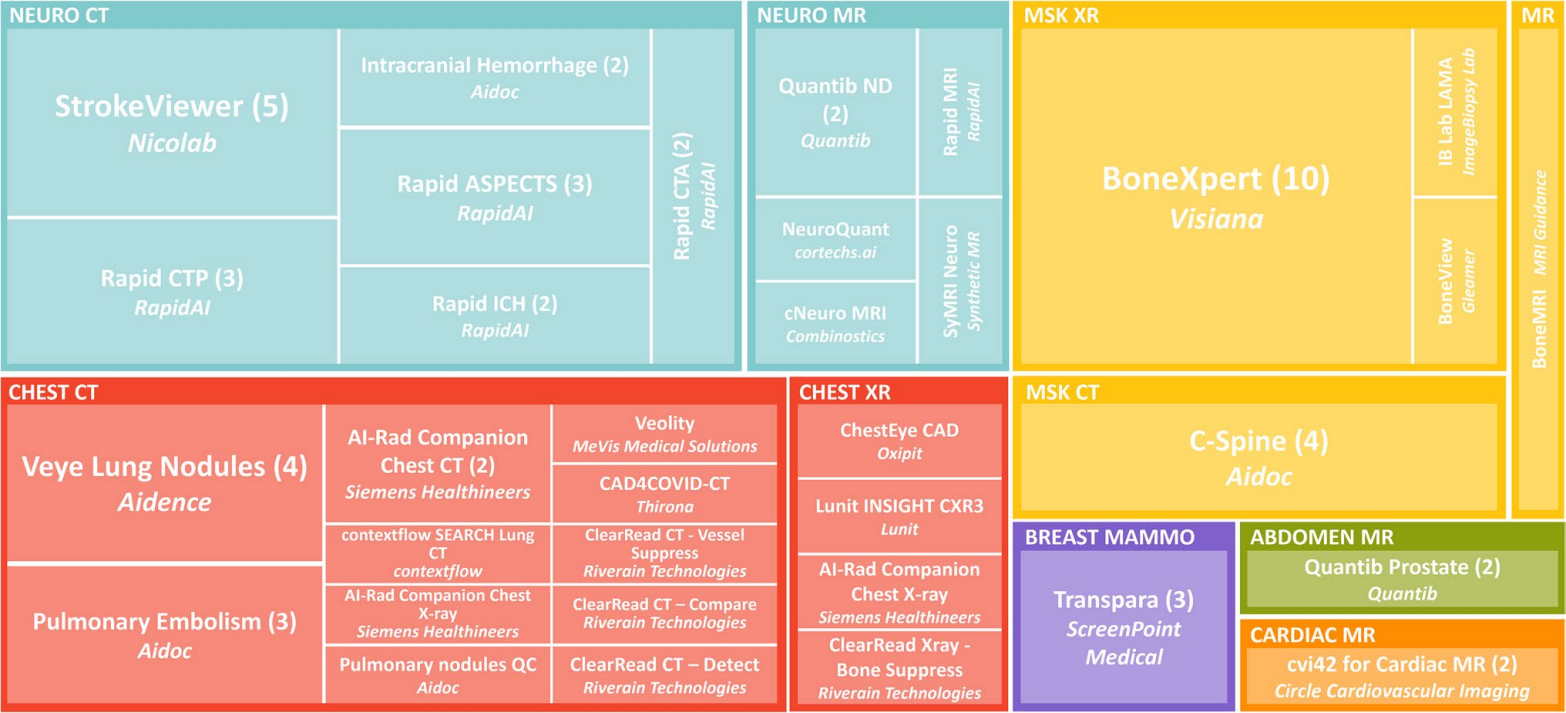
Commercial AI products used in radiology departments in the Netherlands according to survey results from spring 2022. Product names are provided with the company name in italic. The number represents the number of implementations. If no number was given there was one implementation

In 2022, AI is mostly used to support chest CT analysis (nodule detection, pulmonary embolism detection, covid severity) (*n* = 17), neuro CT analysis (large vessel occlusion detection, intracranial hemorrhage detection, ASPECTS, CTP) (*n* = 17), followed by musculoskeletal radiograph analysis (bone age prediction, fracture detection, and automated extremity measurements) (*n* = 12). Figure 2 provides an overview of the products adopted according to the latest survey (2022).

**Fig. 3:**
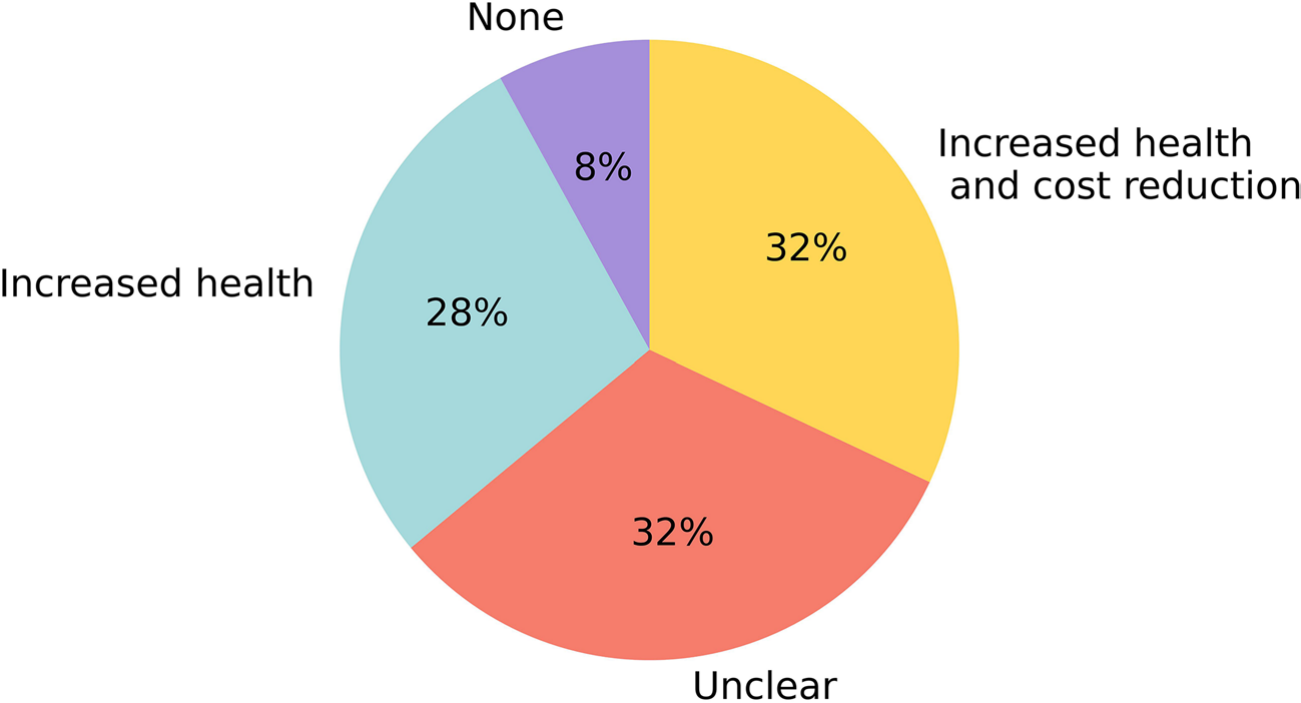
Self-reported value of AI products in use by the respondent’s departments in 2022. Answers are provided by the representatives of the centers using AI in 2022

Figure 3 shows the considered clinical value of AI in 2022. The added value was not necessarily researched or proven, but mostly a subjective judgment of the responder. From all respondents that used AI clinically in their department, 28% stated that the implemented AI products realized health improvement and 32% assumed both health improvement and cost savings. No one reported cost savings alone. Value was considered unclear for 32% of the respondents and 8% said to experience no added value.

### Obstacles and budget for implementation

**Fig. 4:**
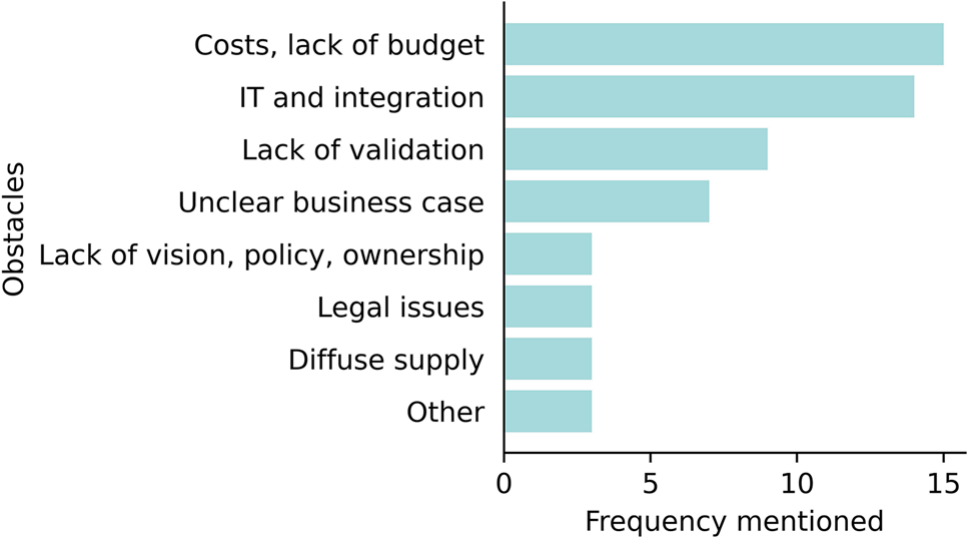
Main obstacles experienced for the purchase, validation, and implementation of AI tools in clinical practice as responded in 2022. Bars represent the frequency of the item mentioned by respondents. Multiple items per respondent were possible

In all three editions, the most frequently mentioned obstacles regarding AI procurement, validation, and implementation were financial difficulties (high costs and lack of budget) (*n* = 15 in 2022) and IT and integration issues (lack of internal resources or dissatisfactory integration possibilities) (*n* = 14 in 2022). Legal issues seem to have subsided over time (*n* = 6 in 2020 vs *n* = 3 in 2022) and lack of validation became a more important obstacle (*n*=6 in 2020 vs *n*=9 in 2022). Figure 4 shows the frequency of obstacles mentioned in the 2022 questionnaire.

**Fig. 5:**
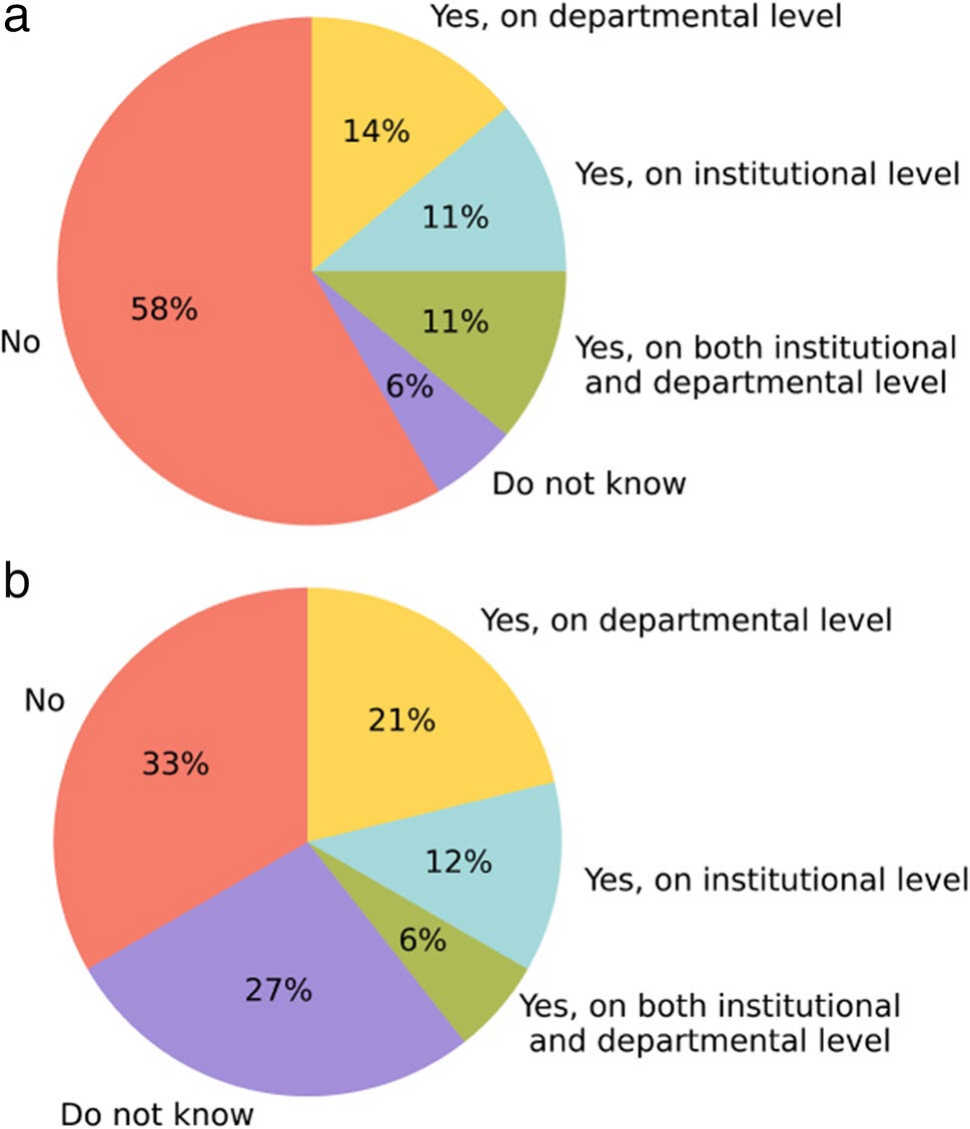
Budget reserved for purchasing AI software in 2021 (**a**) and 2022 (**b**)

In both 2021 and 2022, 13 respondents mentioned that their center had a budget reserved for radiology AI purchases in the coming year (36% in 2021, 39% in 2022). The remainder had no budget or did not know whether there was a budget reserved. Financial resources originate from the institutional level, the radiology department, or both. The source slightly shifted to the departmental level in 2022, as shown in Fig. 5.

## Discussion

The adoption of commercial AI products in radiology departments in the Netherlands has steadily increased over the past three years, encompassing at least one out of three centers in 2022 as opposed to one out of five in 2020. The academic and teaching hospitals are leading the way. Especially the number of implementations and diversity of AI software products have rapidly increased showing that AI-adopted departments have been scaling up its use. In 2022, several centers discontinued the use of some AI products; however, the majority (60%) report that AI is bringing added value to their clinical practice in the form of health benefits. The most commonly adopted AI products are for lung nodule detection on CT, stroke diagnosis, and bone age prediction. The major obstacles to AI adoption remain financial and IT-related issues.

In 2020 the ACR Data Science Institute, held a survey across the United States to examine the use of AI software in clinical practice [5]. They found that about a third of the radiologists were using AI, mostly from larger practices. Even though they did not identify this on a per-practice basis, these results seem to be congruent with our findings in the Netherlands. A striking difference is the most common AI use case. In the US this was software for mammography analysis, which has most likely been influenced by the reimbursement of computer-aided detection (CAD) for mammography since the start of this century. This also directly demonstrates the impact that reimbursement has on the adoption of AI [9]. Lack of budget and high purchase and implementation costs have remained the major obstacles holding back AI adoption, especially in general hospitals. Interestingly, no single center reported that their AI products were cost-reducing alone, showing that the goal of increased health is more present in the current offerings and implementations. This may encumber adoption as monetizing health gains is not as straightforward as e.g. direct efficiency improvements in the department itself.

Considering the then available CE-marked products, only 17% (34/202) of them are being deployed in the Netherlands. Important to note, is that 18 of the 68 implementations (29%) were products originating from Dutch AI vendors. With 8 AI companies marketing 13 products, the Netherlands is an above-average global market player. It is probable that these companies stimulate the adoption of AI in clinical practice and have a considerable impact on the use cases being addressed in our country. The fact that the Netherlands is a high-income country and has a vivid healthcare (AI) technology market, results may not directly extrapolate to other countries. Both the beneficial use cases of AI (e.g. more autonomous AI in low-income countries) as well as the adoption grade (through availability of resources and presence of technology push) may differ.

In this study, reported products that were not on www.AIforRadiology.com were excluded from the analysis. Similar to what was found in the ACR survey, there is not a shared view on what is considered AI software and what is not. This approach was chosen to have a common ground and make the study reproducible and may have led to a slight underrepresentation of AI use. To illustrate the effect, without this exclusion criterion, 25 hospital organizations (instead of 23) would be having 80 implementations (instead of 68) of 45 different tools (instead of 34) in 2022. Excluded products were mostly traditional CAD tools, device-embedded software, and post-processing or workflow software.

A limitation of this research is the response rate and possible bias. We did not manage to get insights on the use of AI from each center in the Netherlands. More engagement is seen from the centers using AI, while we notice dropout after the first year by centers not using AI yet. Also, the single representatives per center may not have had the overview of the whole department influencing the quality of the data and may report more positively on AI as they are usually the early adapters and project leads. The latter may have had a large influence on the results of the considered added value of AI in clinical practice. As many clinical centers do not thoroughly research the impact of their AI implementations, this should be considered a subjective judgement. The results do illustrate the general sentiment of the respondents on their experiences with AI, which based on their answers is positive.

In the past years, the adoption of radiology AI in the Netherlands has been growing. But also, the first signs of stagnation are emerging as implementations are discontinued, because of lack of budget or disappointing experiences. This is not necessarily to be worried about: these are common signs of a new market impending a mature state [10]. With the increase of real-world evidence, the added value of AI tools will become more apparent, separating the wheat from the chaff. This in turn should help to overcome the adoption hurdles and make AI in radiology mainstream.

### Supplementary information

ESM 1 (PNG 169 kb)

ESM 2 (PDF 163 kb)

## Abbreviations

AI: Artificial intelligence
CE: Conformité Européene
CAD: Computer-aided detection

## Declarations

### Guarantor

The scientific guarantor of this publication is Bram van Ginneken.

### Statistics and biometry

No complex statistical methods were necessary for this paper.

### Methodology

- prospective
- cross-sectional study
- multicenter study
