# Supplementary figures and images for: Clinical use of artificial intelligence products for radiology in the Netherlands between 2020 and 2022
Source: Eur Radiol. 2023 Jul 29;34(1):348–54. doi: 10.1007/s00330-023-09991-5 (PMC10791748; doi:10.1007/s00330-023-09991-5)

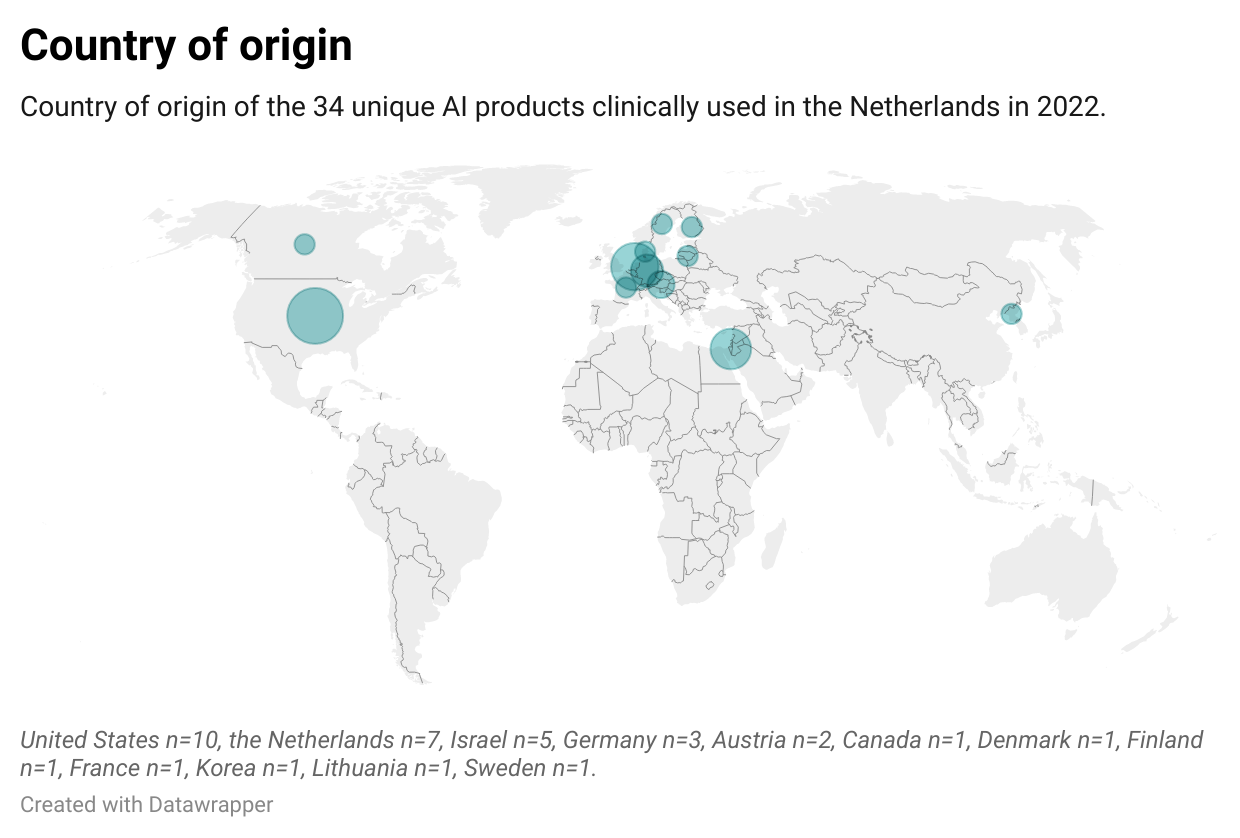

Supplement: Supplementary file 1 — (PNG 169 kb) [file 330_2023_9991_MOESM1_ESM.png]
