# Supplementary material for: Clinical use of artificial intelligence products for radiology in the Netherlands between 2020 and 2022
Source: Eur Radiol. 2023 Jul 29;34(1):348–54. doi: 10.1007/s00330-023-09991-5 (PMC10791748; doi:10.1007/s00330-023-09991-5)
Supplement: Supplementary file 2 — (PDF 163 kb) [file 330_2023_9991_MOESM2_ESM.pdf]

Inventarisatie klinisch AI gebruik in de radiologie

Met deze korte vragenlijst inventariseren we welke radiologie afdelingen AI software geïmplementeerd hebben. Met AI software bedoelen we software die gebruik maakt van machine learning of geavanceerde beeldanalyse technieken ten behoeve van de dagelijkse werkzaamheden van de radioloog of nucleair geneeskundige.

Resultaten van vorig jaar zijn te vinden op: https://www.radiologen.nl/contactpersonen-artificial-intelligence-ai-ai-netwerk

*Required

1. Naam *

____

2. E-mailadres *

   We gebruiken uw e-mailadres alleen in kader van dit onderzoek en wordt niet verstrekt aan derden.

____

3. Ziekenhuis organisatie *

Mark only one oval.

- Admiraal De Ruyter Ziekenhuis
- Albert Schweitzer Ziekenhuis
- Alrijne Zorggroep
- Amphia Ziekenhuis
- Amsterdam UMC
- Antoni van Leeuwenhoek
- Antonius Zorggroep
- BovenIJ Ziekenhuis
- Bravis Ziekenhuis
- Canisius-Wilhelmina Ziekenhuis
- Catharina Ziekenhuis
- Deventer Ziekenhuis
- Diakonessenhuis
- Dijklander Ziekenhuis
- Elisabeth-TweeSteden
- Elkerliek Ziekenhuis
- Erasmus Medisch Centrum
- Flevoziekenhuis
- Franciscus Gasthuis & Vlietland Groep
- Gelre Ziekenhuizen
- Groene Hart Ziekenhuis
- Haaglanden Medisch Centrum
- Het Van Weel-Bethesda Ziekenhuis
- IJsselland Ziekenhuis
- Ikazia Ziekenhuis
- Isala klinieken
- Jeroen Bosch Ziekenhuis
- Laurentius Ziekenhuis
- Leids Universitair Medisch Centrum
- Maasstad ziekenhuis
- Maastricht UMC+
- Martini Ziekenhuis

- Máxima Medisch Centrum
- Meander Medisch Centrum
- Medisch Spectrum Twente
- Nij Smellinghe
- Noordwest Ziekenhuisgroep
- Ommelander Ziekenhuis Groep
- Onze Lieve Vrouwe Gasthuis
- Pantein
- Prinses Máxima Centrum voor kinderoncologie
- Rijnstate Ziekenhuis
- Rivas Zorggroep
- Rode Kruis Ziekenhuis
- Santiz
- Saxenburgh Groep
- Spaarne Gasthuis
- Spijkenisse Medisch Centrum
- St. Anna Zorggroep
- St. Antonius Ziekenhuis
- St. Jans Gasthuis
- Stichting Reinier Haga Groep
- Tergooiziekenhuizen
- Treant Zorggroep
- Universitair Medisch Centrum Groningen
- Universitair Medisch Centrum St. Radboud
- Universitair Medisch Centrum Utrecht
- VieCuri Medisch Centrum
- Wilhelmina Ziekenhuis Assen
- Zaans Medisch Centrum
- Ziekenhuis Amstelland
- Ziekenhuis Bernhoven
- Ziekenhuis De Gelderse Vallei
- Ziekenhuis Rivierenland
- Ziekenhuis St. Jansdal
- Ziekenhuisgroep Twente
- Zorgpartners Friesland

- ZorgSaam Zeeuws-Vlaanderen
- Zuyderland Medisch Centrum
- Anders

4. Functie/Rol *

____

5. Is de wens er om op de afdeling radiologie AI software te implementeren? *

Mark only one oval.

- Ja, we gebruiken het al
- Ja, in de nabije toekomst *Skip to question 7*
- Misschien *Skip to question 7*
- Nee, voorlopig nog niet *Skip to question 11*
- Nee, niet meer

Waarde

6. Levert het klinische gebruik van AI... *

Mark only one oval.

- Gezondheidswinst
- Kostenbesparing
- Beiden
- Geen
- Other: _______________________

AI gebruik

7. Welke AI producten voor de radiologie gebruikt uw afdeling in de dagelijks klinische praktijk?

   Graag product en bedrijfsnaam. Zie www.AIforRadiology.com voor een overzicht.

8. Zijn er AI producten waarvan het klinisch gebruik is gestaakt?

   Graag product, bedrijfsnaam en reden.

9. Maakt uw afdeling gebruik van een AI marktplaats/platform? Zo ja, welke?

   Denk aan bijvoorbeeld Amplifier Store (Sectra), IntelliSpace AI Workflow Suite (Philips), Eureka Clinical AI Platform (TeraRecon), etc.

10. Met welke AI producten (niet eerder genoemd) doet uw afdeling onderzoek of heeft u onderzoek mee gedaan?

    Graag product en bedrijfsnaam.

Als laatste...

11. Wat zijn de moeilijkheden waar u tegen aan loopt bij de aanschaf/validatie/implementatie van AI software?

____

____

____

____

____

12. Is er voor 2022 budget gereserveerd voor de aanschaf van AI? *

Tick all that apply.

- [ ] Ja, op afdelingsniveau
- [ ] Ja, op ziekenhuisniveau
- [ ] Nee
- [ ] Weet ik niet
- [ ] Other: ______________________________

13. Overige vragen of opmerkingen

____

14. Ik ga ermee akkoord deze gegevens te delen met de NVvR en betreffende onderzoekers van het Radboudumc. *

De antwoorden op de vragen worden geanonimiseerd gebruikt voor academische doeleinden. Daarnaast wordt (een deel van) de informatie, met benoeming van het ziekenhuis, beschikbaar gemaakt voor NVvR leden op de NVvR website ter bevordering van kennisdeling en samenwerking. Mochten er specifieke vragen of bezwaren zijn, neem dan contact op met Kicky van Leeuwen, kicky.vanleeuwen@radboudumc.nl

Tick all that apply.

- [ ] Akkoord

This content is neither created nor endorsed by Google.

Google Forms
